# The Impact of Molecular Characteristics on the Efficacy of Frontline Immune Checkpoint Inhibitor Therapy in Patients with Metastatic Melanoma

**DOI:** 10.3390/cancers18172768

**Published:** 2026-08-26

**Authors:** Seo Yoon Jang, Minsuk Kwon, Jeeyun Lee, Seung Tae Kim

**Affiliations:** Division of Hematology-Oncology, Department of Medicine, Samsung Medical Center, Sungkyunkwan University School of Medicine, Seoul 06351, Republic of Korea; sy3.jang@samsung.com (S.Y.J.); minsuk.dr.kwon@samsung.com (M.K.); jyunlee@skku.edu (J.L.)

**Keywords:** melanoma, immune checkpoint inhibitor, molecular subtype

## Abstract

**Background:** Metastatic melanoma in East Asian populations is enriched for acral and mucosal subtypes and harbors a distinct molecular landscape compared with Western cutaneous melanoma. However, data on the efficacy of first-line immune checkpoint inhibitor (ICI) therapy and on the impact of molecular characteristics on treatment effect in this population remain limited. We aimed to characterize the genomic landscape of an East Asian metastatic melanoma cohort and to evaluate the predictive values of molecular markers for first-line ICI therapy. **Methods:** This study included 135 patients with metastatic melanoma who received first-line ICI at Samsung Medical Center between January 2022 and December 2025. Of these, 108 with paired next-generation sequencing (NGS) data were included in the molecular analysis. Survival outcomes were estimated by the Kaplan–Meier method, and the prognostic value of molecular subtype was assessed using univariable and multivariable Cox proportional hazards models. **Results:** Mucosal melanoma was the most common primary site (58, 43.0%), followed by acral melanoma (31, 23.0%) and cutaneous melanoma (25, 18.5%); 4 (3.0%) had uveal melanoma and 17 (12.6%) had melanoma of other or unknown primary origin. The overall objective response rate to first-line ICI was 37.8% (51 of 135), and median progression-free survival (PFS) was 6.2 months (95% confidence interval [CI] 4.0–9.6). Using the hierarchical classification, 108 patients were classified into five molecular subtypes: *BRAF*-altered (*n* = 17, 15.7%), *RAS*-altered (*n* = 17, 15.7%), *KIT*-altered (*n* = 15, 13.9%), and *NF1*-altered (*n* = 7, 6.5%), and quadruple–wild-type (*n* = 52, 48.1%). TMB-high status (10.2%) showed no significant association with outcomes. Molecular subtype was significantly associated with PFS (log-rank *p* = 0.009). After multivariable adjustment, *BRAF* fusion (hazard ratio [HR] 5.05, 95% CI 1.70–14.98, *p* = 0.003) and *KIT*-altered status (HR 2.91, 95% CI 1.46–5.77, *p* = 0.002) emerged as independent adverse prognostic factors, whereas *BRAF* V600 single-nucleotide variants did not differ significantly from quadruple–wild-type. **Conclusions:** In this East Asian metastatic melanoma cohort, *BRAF* fusion and *KIT* alterations were independent adverse prognostic factors for first-line ICI. These findings suggest that *BRAF* fusion and *KIT*-altered tumors may represent distinct subgroups that warrant further investigation.

## Simple Summary

Melanoma is a serious skin cancer, and in East Asian patients it frequently arises at acral sites or on internal mucosal surfaces rather than on sun-exposed skin. These forms tend to respond less well to immunotherapy, the current standard first-line treatment, yet it remains unclear which tumors benefit least. We studied 135 East Asian patients with metastatic melanoma treated with first-line immunotherapy and analyzed the genetic features of their tumors. Tumors carrying *BRAF* gene fusions and *KIT* alterations were associated with markedly poorer outcomes. Identifying these subgroups may help clinicians recognize patients unlikely to benefit from immunotherapy alone and guide the development of more effective treatment strategies for this understudied population.

## 1. Introduction

Melanoma accounts for the majority of skin cancer–related deaths despite representing only a small fraction of cutaneous malignancies, and its global incidence continues to rise [1]. Although most melanomas in Western populations arise from melanocytes of sun-exposed skin, melanoma can also originate from acral sites or mucosal surfaces [2,3]. In East Asian populations, including Korean patients, acral and mucosal subtypes together account for approximately half of all metastatic melanoma cases, in contrast to fewer than 10% in cohorts of European ancestry [4,5,6].

The therapeutic landscape of metastatic melanoma has been transformed by immune checkpoint inhibitors (ICIs); anti–PD-1 monotherapy, alone or combined with anti–CTLA-4, is now a standard first-line systemic therapy [7,8]. However, the pivotal trials that established this paradigm enrolled predominantly patients with cutaneous melanoma of Western ancestry. A consistent finding in subsequent real-world and prospective Asian series is that objective response rates (ORRs) and progression-free survival (PFS) with anti–PD-1 monotherapy in acral and mucosal melanoma are approximately half those reported in cutaneous disease [4,5,9,10]. The efficacy of first-line ICI in acral- and mucosal-predominant cohorts therefore remains an important question, and few studies have reported outcome data integrated with molecular profiling in such populations.

At the molecular level, melanoma comprises distinct subtypes rather than a single disease [11,12]. The Cancer Genome Atlas proposed a four-class taxonomy for cutaneous melanoma based on the predominant mitogen-activated protein kinase (MAPK) pathway driver: *BRAF*-, *RAS*-, *NF1*-mutant, and triple–wild-type [13]. In acral and mucosal melanoma, however, *BRAF* V600 mutations are markedly less frequent, whereas *KIT* mutations and amplifications represent a clinically actionable driver; non-cutaneous–enriched cohorts, including the present analysis, have therefore increasingly treated *KIT*-altered tumors as a distinct class extending this taxonomy [14,15,16]. These subtypes may also influence ICI outcomes [17,18], yet subtype-specific prognostic data come almost exclusively from Western cutaneous cohorts and have been inconsistent [19,20].

In the present study, we evaluated the efficacy of first-line ICI in a Korean cohort of metastatic melanoma patients in which acral and mucosal histologies predominated. By integrating targeted next-generation sequencing (NGS), we also characterized the molecular landscape, defined its association with the primary site, and assessed whether molecular subtype is an independent prognostic factor for PFS on first-line ICI.

## 2. Materials and Methods

### 2.1. Patient Enrollment

This study included 135 patients with metastatic melanoma who received first-line ICI at Samsung Medical Center between January 2022 and December 2025. Eligible regimens included the anti–PD-1 monotherapy pembrolizumab or nivolumab. Patients were eligible if they were aged 18 years or older, had pathologically confirmed melanoma, presented with stage IV disease, and had radiographically measurable disease at the start of palliative first-line ICI. Patients receiving ICI in the second-line or later setting and those without a recorded best-response assessment at the time of data cut-off were excluded.

Demographic data, clinical data, and laboratory parameters were extracted from electronic medical records. The study was approved by the Institutional Review Board of Samsung Medical Center (IRB no. 2026-06-114) and was conducted in accordance with the Declaration of Helsinki. The requirement for informed consent was waived due to the retrospective nature of the study.

### 2.2. Next-Generation Sequencing

Targeted NGS was performed on archived tumor tissue or plasma circulating tumor DNA (ctDNA), with the choice of medium based on tissue availability and clinical context. Tumor tissue libraries were prepared from formalin-fixed, paraffin-embedded biopsy specimens using the TruSight Oncology 500 (TSO500) DNA/RNA NextSeq Kit (Illumina, San Diego, CA, USA) or the Oncomine Comprehensive Assay Plus (Thermo Fisher Scientific, Waltham, MA, USA) according to the manufacturers’ protocols. For plasma sequencing, cell-free DNA was extracted from 8 mL of plasma, and libraries were prepared using the TSO500 ctDNA assay (Illumina), a 1.94 Mb hybrid capture–based panel covering 523 genes. Sequencing was performed on an Illumina NovaSeq 6000 instrument (Illumina, San Diego, CA, USA).

Bioinformatic analysis followed the manufacturer’s pipeline for each platform, including DRAGEN TSO500 ctDNA Analysis Software v1.1.0 (Illumina) for plasma samples. Single-nucleotide variants (SNVs), small insertions and deletions (indels), copy-number variants, and gene fusions were called; SNVs and indels were retained at a variant allele frequency ≥ 2% for tissue and at the platform-specific limit of detection for plasma, and copy numbers > 4 or <1 were considered amplifications or deletions, respectively. Variants were annotated using the Ensembl Variant Effect Predictor and classified per the joint Association for Molecular Pathology/American Society of Clinical Oncology/College of American Pathologists guidelines; only Tier I and Tier II variants were retained for analysis. Tumor mutational burden (TMB) and microsatellite instability (MSI) were computed by the TSO500 pipeline; TMB-high was defined as ≥10 mutations per megabase (mut/Mb), and MSI status was classified as microsatellite stable (MSS), MSI-high (MSI-H), or indeterminate.

### 2.3. Molecular Subtype Classification

Patients with sequencing data were assigned to one of five mutually exclusive molecular subtypes based on the predominant driver alteration, extending the four-class genomic taxonomy of cutaneous melanoma proposed by The Cancer Genome Atlas to include a fifth, *KIT*-altered class given the high prevalence of *KIT* alterations in acral and mucosal melanoma. When more than one driver alteration was present in the same patient, classification followed a fixed hierarchy: *BRAF*-altered > *RAS*-altered > *NF1*-altered > *KIT*-altered > quadruple–wild-type.

The *BRAF*-altered subtype was defined by the presence of a Tier I/II *BRAF* V600 SNV or a *BRAF* gene fusion; *BRAF* amplification alone was not considered subtype-defining, since it is generally considered a resistance mechanism rather than a primary oncogenic driver. The *RAS*-altered subtype was defined by a Tier I/II hotspot SNV in *NRAS*, *KRAS*, or *HRAS* at codons G12, G13, or Q61. The *NF1*-altered subtype was defined by a Tier I/II truncating, splice-site, or other loss-of-function SNV in *NF1*, or by *NF1* deletion. The *KIT*-altered subtype was defined by a Tier I/II *KIT* SNV within exon 11, 13, or 17, or by *KIT* amplification, both of which are recognized oncogenic drivers in acral and mucosal melanoma. Patients with no qualifying alteration in any of the four genes were classified as quadruple–wild-type. Non-V600 *BRAF* variants, non-hotspot *RAS* variants, and *BRAF*, *NRAS*, or *KRAS* amplifications did not satisfy the subtype-defining criteria and were assigned to the quadruple–wild-type group when no other driver was present.

Because *BRAF* V600 SNVs and *BRAF* fusions have been suggested to differ in clinical behavior on ICI therapy, the *BRAF*-altered group was additionally divided into *BRAF* V600 and *BRAF* fusion subgroups in both the univariable and multivariable analyses.

### 2.4. Treatment and Response Assessment

First-line ICI consisted of pembrolizumab or nivolumab at standard label doses, continued until disease progression, unacceptable toxicity, or patient or physician decision. During the study period, both pembrolizumab and nivolumab were reimbursed and equally available for first-line treatment of advanced melanoma in Korea; the choice between the two agents was therefore based on physician preference rather than on drug availability or on tumor- or patient-related characteristics. Tumor response was assessed by the treating physician according to the Response Evaluation Criteria in Solid Tumors (RECIST) version 1.1. The best overall response was recorded as complete response (CR), partial response (PR), stable disease (SD), or progressive disease (PD). The ORR was defined as the proportion of patients achieving CR or PR, and the disease control rate (DCR) as the proportion achieving CR, PR, or SD as the best overall response. PFS was calculated from the date of first ICI administration to the date of first radiographically confirmed disease progression or death from any cause, whichever occurred first. Overall survival (OS) was calculated from the date of first ICI administration to the date of death from any cause. Patients without an event were censored at the date of last follow-up. The data cut-off date was 31 May 2026.

### 2.5. Statistical Analysis

Continuous variables were presented as median with interquartile range (IQR), and categorical variables as count with percentage. PFS and OS were estimated using the Kaplan–Meier method with 95% confidence intervals (CIs), and between-group differences were tested with the log-rank test. ORR and DCR were compared using the chi-square test or Fisher’s exact test, as appropriate. The association between primary site and molecular subtype was evaluated using Fisher’s exact test. Hazard ratios (HRs) with 95% CIs for PFS were estimated by Cox proportional hazards regression, and the proportional hazards assumption was assessed using scaled Schoenfeld residuals. Variables that were statistically significant in the univariable analysis or considered clinically important were entered into the multivariable Cox model, with quadruple–wild-type as the reference category; within the *BRAF*-altered group, *BRAF* V600 and *BRAF* fusion were modeled as separate categories. The neutrophil-to-lymphocyte ratio (NLR) was dichotomized at a value of 3, in line with the conventional threshold used in melanoma ICI cohorts [21]. In sensitivity analyses, the model was refitted with prior adjuvant ICI as an additional covariate, and a Firth penalized Cox model was fitted to assess potential small-sample and separation bias. In addition, a Cox model stratified by primary site was fitted to assess confounding between molecular subtype and primary site. Because of the limited number of OS events, multivariable analysis was not performed for OS. All statistical tests were two-sided, and a *p* value less than 0.05 was considered statistically significant. Analyses were performed using R Statistical Software (version 4.4.1, Foundation for Statistical Computing, Vienna, Austria).

## 3. Results

### 3.1. Patient Characteristics

A total of 200 melanoma treatment records were screened for eligibility. After exclusion of 16 records for no measurable lesion at the start of ICI and 49 for second-line or later therapy, the final cohort comprised 135 patients (Figure S1, CONSORT). NGS was available for 108 patients (80.0%).

Baseline characteristics of the 135 patients are summarized in Table 1. The median age was 64.7 years (IQR, 57.2–73.3), with 64 patients (47.4%) aged 65 years or older, and 74 patients (54.8%) were male. 116 patients (85.9%) received pembrolizumab, while 19 patients (14.1%) received nivolumab monotherapy. Seven patients (5.2%) had received prior ICI in the adjuvant setting before initiating palliative first-line therapy.

Reflecting the distinct epidemiology of melanoma in East Asia, acral and mucosal histologies together accounted for two-thirds of the cohort. Mucosal melanoma was the most common primary site (58, 43.0%), followed by acral melanoma (31, 23.0%) and cutaneous melanoma (25, 18.5%); 4 (3.0%) had uveal melanoma and 17 (12.6%) had melanoma of other or unknown primary origin.

### 3.2. Efficacy Outcomes

**Figure 1:**
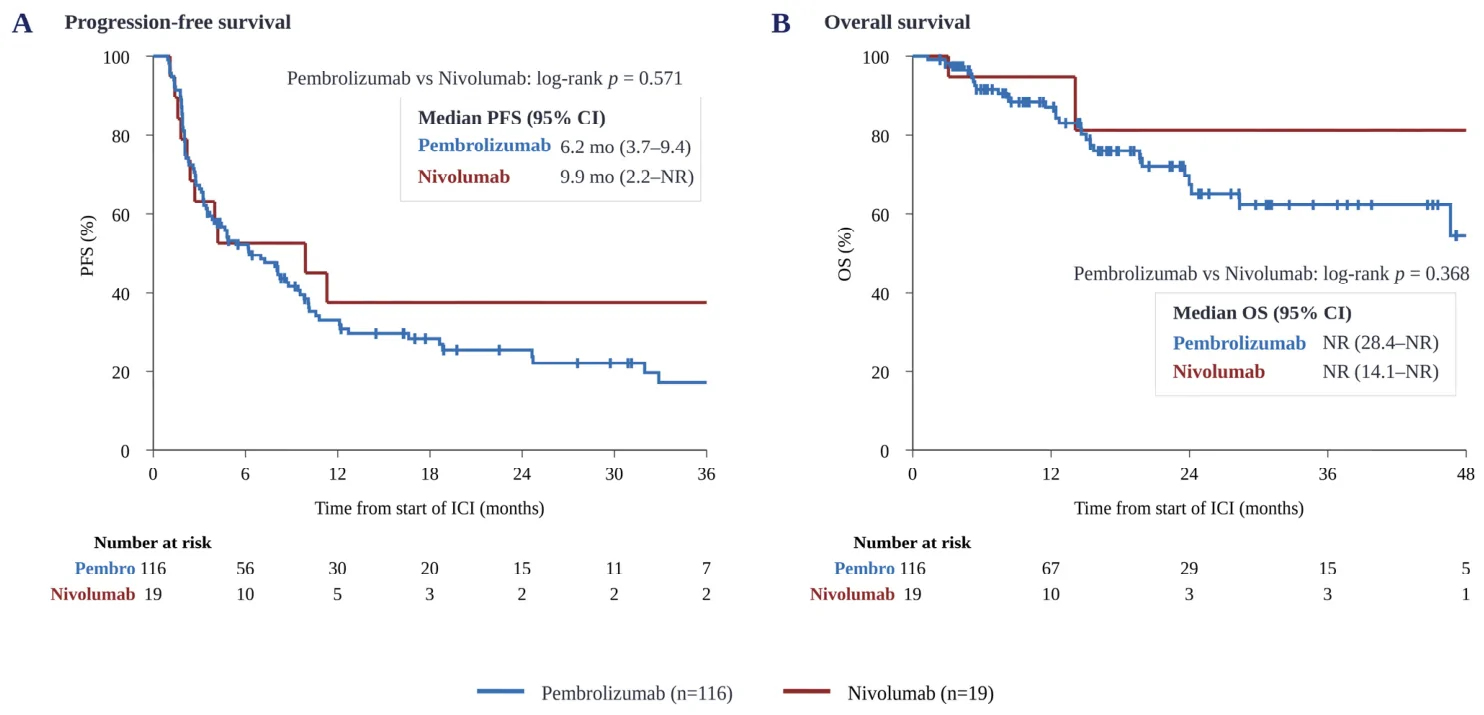
Kaplan–Meier curves for progression-free and overall survival. (**A**) Progression-free survival for pembrolizumab versus nivolumab (log-rank *p* = 0.571); (**B**) Overall survival for pembrolizumab versus nivolumab (log-rank *p* = 0.368). ICI, immune checkpoint inhibitor; PFS, progression-free survival; OS, overall survival; CI, confidence interval; NR, not reached.

Efficacy outcomes by ICI regimen are summarized in Table 2. The best overall response was CR in 6 patients (4.4%), PR in 45 (33.3%), SD in 41 (30.4%), and PD in 43 (31.9%). The ORR was 37.8% (95% CI, 30.0–46.2), and the DCR was 68.1% (95% CI, 59.9–75.4). The median PFS was 6.2 months (95% CI, 4.0–9.6), with 6-, 12-, and 24-month PFS estimates of 52.4%, 33.8%, and 27.1%, respectively. The median OS was not reached (95% CI, 46.6–not reached), with 12- and 24-month OS estimates of 88.1% and 68.9%, respectively (Figure 1).

Pembrolizumab was the predominant first-line regimen (*n* = 116), with an ORR of 39.7% and a median PFS of 6.2 months. Between pembrolizumab and nivolumab monotherapy, no statistically significant difference was observed in either PFS (log-rank *p* = 0.571) or OS (log-rank *p* = 0.368).

### 3.3. Molecular Landscape

Among the 108 patients with NGS data, 88 (81.5%) were profiled by tumor tissue sequencing and 20 (18.5%) by plasma ctDNA sequencing. The median TMB was 4.7 mut/Mb (IQR, 2.8–7.2), and 11 patients (10.2%) had a TMB-high tumor. MSI status was MSS in 97 patients (89.8%) and indeterminate in 11 (10.2%), the latter predominantly reflecting ctDNA-based profiling; no patient had an MSI-H tumor.

**Figure 2:**
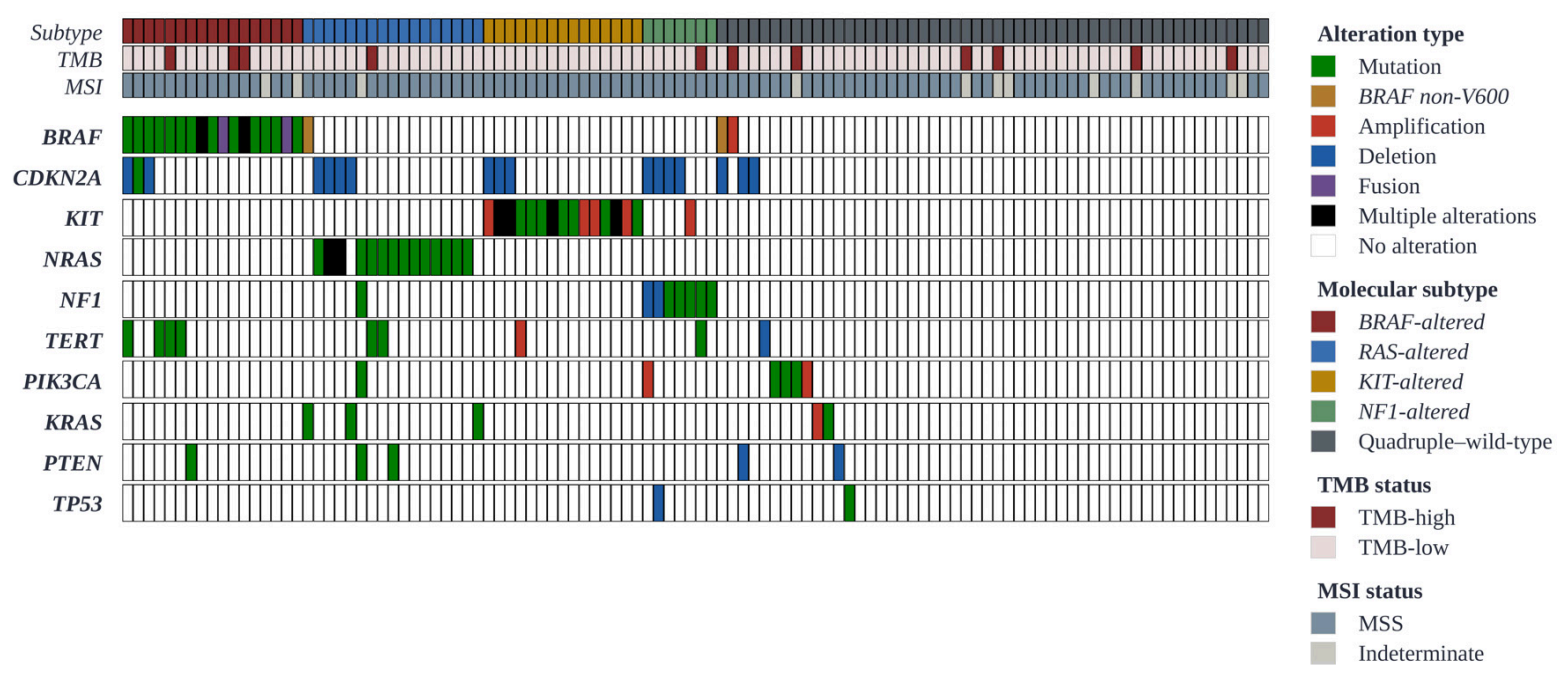
Genomic landscape of melanoma-relevant alterations in the NGS subset (*n* = 108).

Using the hierarchical classification, *BRAF*-altered melanoma was identified in 17 patients (15.7%), *RAS*-altered in 17 (15.7%), *KIT*-altered in 15 (13.9%), and *NF1*-altered in 7 (6.5%); the remaining 52 patients (48.1%) were classified as quadruple–wild-type. Driver-gene alterations, TMB, and MSI status across the cohort, ordered by molecular subtype, are summarized in Figure 2, and the frequencies of co-occurring alterations (*CDKN2A*, *TERT*, *PIK3CA*, *PTEN*, and *TP53*) by subtype are provided in Supplementary Materials Table S1.

Within the *BRAF*-altered subgroup, 13 patients (76.5%) harbored a canonical V600 SNV (V600E in 9, V600K in 2, and V600R in 2), and 4 patients (23.5%) harbored a *BRAF* fusion (AGK–BRAF, NUB1–BRAF, and two MKRN1–BRAF fusions). All four *BRAF* fusions were detected in tumor tissue by the TSO500 DNA/RNA assay.

**Figure 3:**
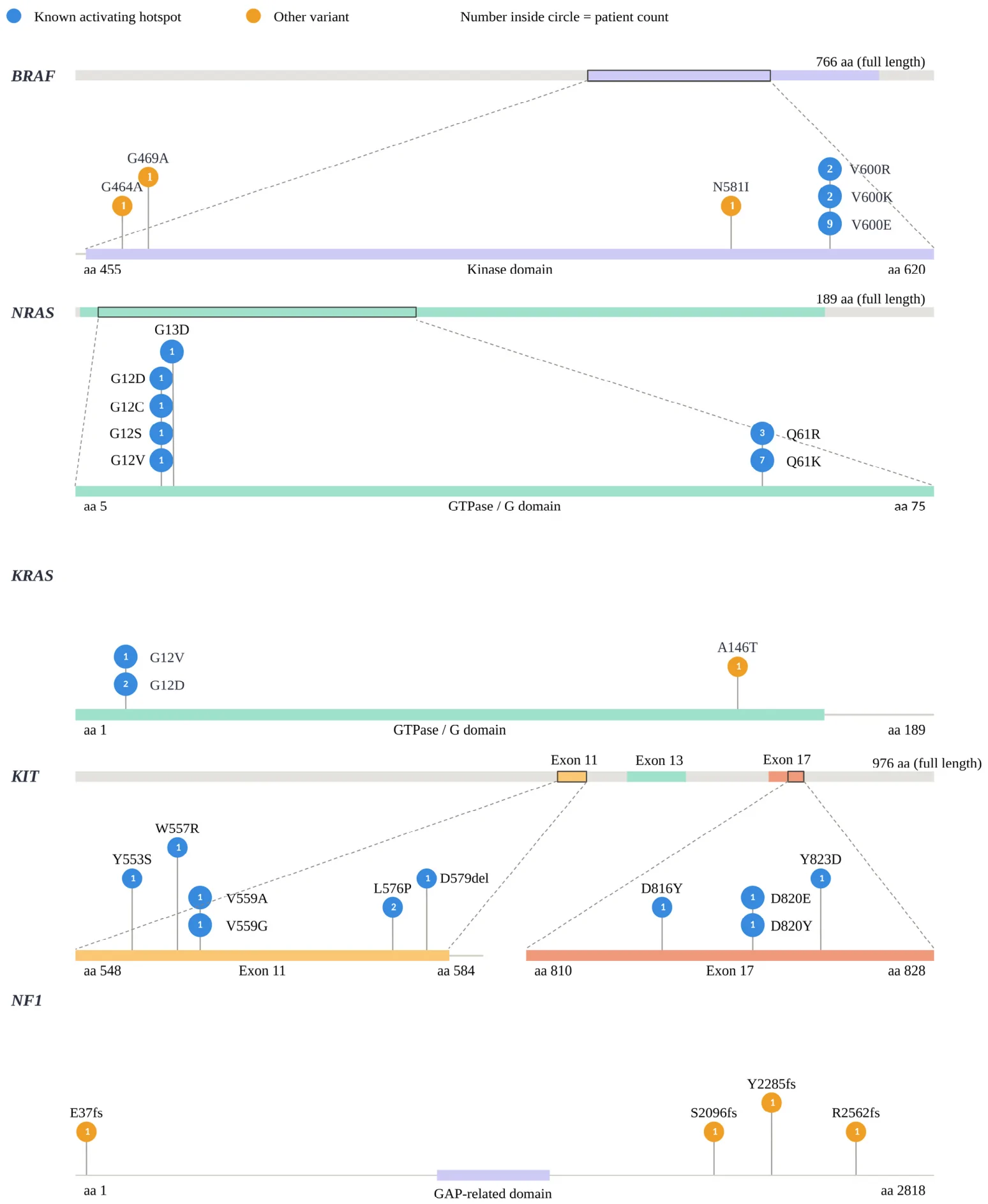
Protein-level distribution of somatic mutations in melanoma-relevant genes. Mutations are shown along the full-length protein for *BRAF*, *NRAS/KRAS*, *KIT*, and *NF1*; mutation-dense regions are magnified.

**Figure 4:**
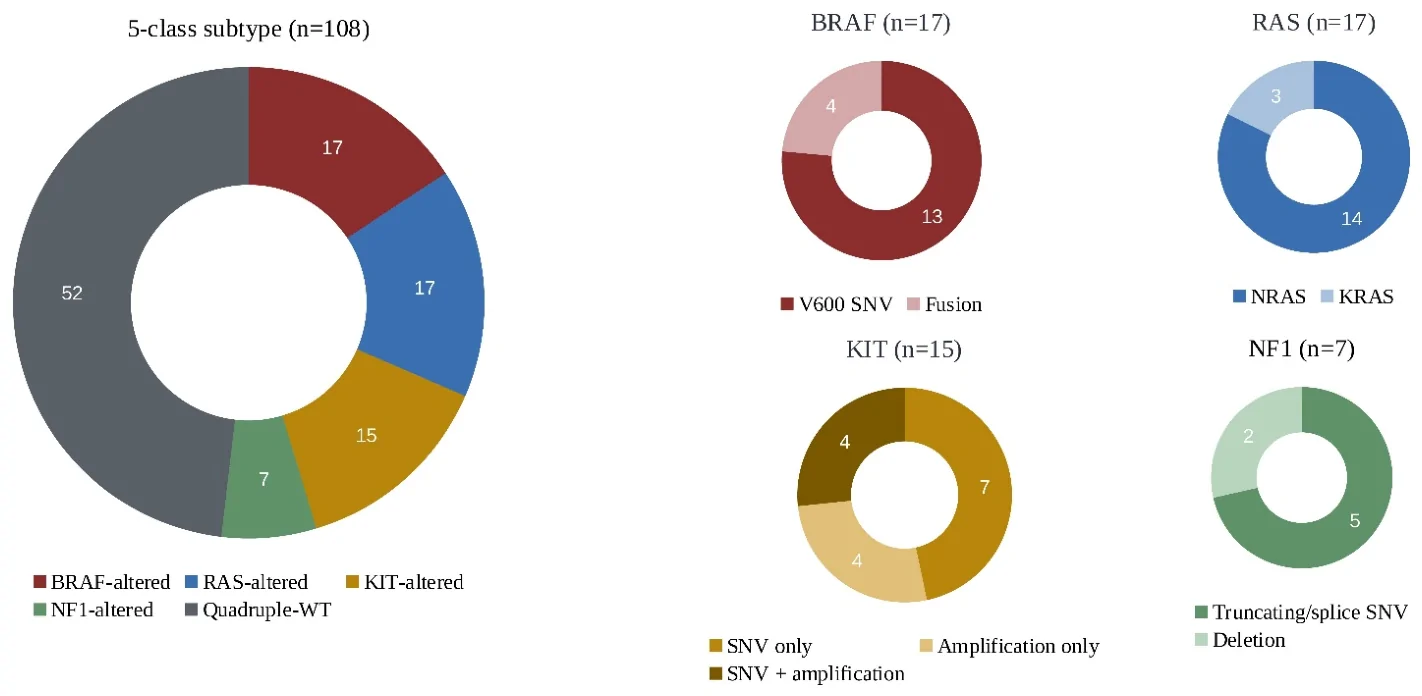
Molecular subtype composition. The central chart shows the 5-class molecular subtype distribution; satellite charts show the alteration-type composition within each altered subtype.

In the *RAS*-altered subgroup, 14 patients harbored an *NRAS* hotspot variant and 3 harbored a *KRAS* hotspot variant. In the *KIT*-altered subgroup, 11 patients carried a hotspot SNV within exon 11 (*n* = 7), exon 13 (*n* = 1), or exon 17 (*n* = 3), and 4 patients harbored *KIT* amplification without a coexisting hotspot variant. The *NF1*-altered subgroup comprised 5 patients with a Tier I/II loss-of-function SNV and 2 with *NF1* deletion. The mutational hotspots and domain-level distribution of recurrent variants in *BRAF*, *NRAS*, *KIT*, and *NF1* are illustrated in Figure 3, and the molecular subtype composition is summarized in Figure 4.

In our cohort, co-occurrence of qualifying driver alterations across more than one of the five categories was uncommon, affecting only 2 of 108 patients (1.9%). One patient harbored an *NRAS* hotspot mutation together with an *NF1* loss-of-function alteration, and the other harbored an *NF1* loss-of-function alteration with *KIT* amplification. No patient harbored qualifying drivers from three or more categories. In these two cases, the hierarchical classification assigned the patient to the higher-priority subtype (*RAS*-altered and *NF1*-altered, respectively). Conversely, 4 tumors (7.7%) assigned to the quadruple-wild-type group carried an alteration that did not meet the subtype-defining criteria: one non-V600 *BRAF* SNV, one non-hotspot *RAS* variant, and two amplification-only cases.

### 3.4. Molecular Subtype-Associated Outcomes

**Figure 5:**
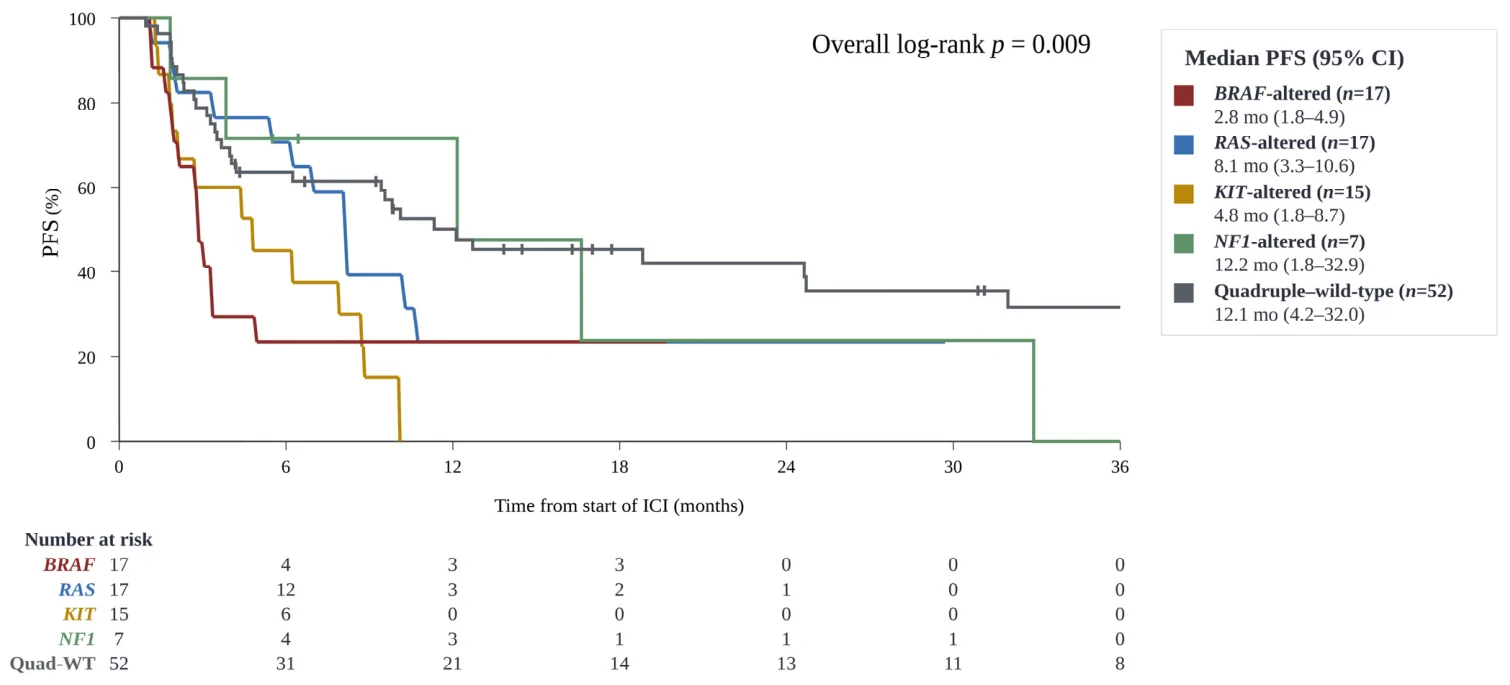
Progression-free survival by molecular subtype.

PFS differed significantly across the five molecular subtypes (log-rank *p* = 0.009). The median PFS was 2.8 months (95% CI, 1.8–4.9) in the *BRAF*-altered subgroup, 4.8 months (95% CI, 1.8–8.7) in the *KIT*-altered subgroup, 8.1 months (95% CI, 3.3–10.6) in the *RAS*-altered subgroup, 12.2 months (95% CI, 1.8–32.9) in the *NF1*-altered subgroup, and 12.1 months (95% CI, 4.2–32.0) in the quadruple–wild-type subgroup (Figure 5). Compared with quadruple–wild-type, both the *BRAF*-altered and *KIT*-altered subgroups had a significantly higher risk of progression (HR 2.50, 95% CI 1.29–4.86, *p* = 0.007; and HR 2.85, 95% CI 1.45–5.61, *p* = 0.002, respectively), whereas no significant difference was observed for the *RAS*-altered (HR 1.49, 95% CI 0.75–2.93, *p* = 0.252) or *NF1*-altered subgroup (HR 1.25, 95% CI 0.49–3.23, *p* = 0.640).

With limited follow-up, OS did not differ significantly across the five subtypes (log-rank *p* = 0.307); the median OS was not reached in the *BRAF*-, *RAS*-, *NF1*-altered, and quadruple–wild-type subgroups, and was 23.6 months (95% CI, 7.4–not reached) in the *KIT*-altered subgroup. ORR by molecular subtype ranged from 23.5% in the *BRAF*-altered subgroup to 71.4% in the *NF1*-altered subgroup (*BRAF*-altered 4/17 [23.5%], *RAS*-altered 9/17 [52.9%], *KIT*-altered 7/15 [46.7%], *NF1*-altered 5/7 [71.4%], quadruple–wild-type 23/52 [44.2%]), although the difference did not reach statistical significance (*p* = 0.231).

The sensitivity analysis within the *BRAF*-altered subgroup showed inferior outcomes relative to quadruple–wild-type, with the effect more pronounced for *BRAF* fusions. The 13 patients with a *BRAF* V600 SNV had a median PFS of 2.8 months and a numerically higher risk of progression compared with quadruple–wild-type that did not reach statistical significance (HR 2.05, 95% CI 0.97–4.36, *p* = 0.061). The 4 patients with a *BRAF* fusion had a median PFS of 2.1 months and a markedly elevated risk of progression (HR 5.20, 95% CI 1.77–15.26, *p* = 0.003), with no objective response observed (0/4).

TMB-high status was not significantly associated with improved outcomes on ICI in this cohort. The median PFS was 9.4 months in the TMB-high subgroup and 8.1 months in the TMB-low subgroup (log-rank *p* = 0.701).

### 3.5. Primary Site and Molecular Subtype Association

Molecular subtype distribution differed markedly across primary sites (*p* < 0.001; Supplementary Materials Figure S2 and Table S2). *BRAF*-altered melanoma was strongly enriched in cutaneous primaries (9/19, 47.4%) and was virtually absent in mucosal primaries (1/50, 2.0%). The odds of *BRAF* alteration were 9.1-fold higher in cutaneous compared with non-cutaneous primaries (odds ratio [OR] 9.11, *p* < 0.001). Conversely, *BRAF* alteration was markedly depleted in mucosal relative to non-mucosal primaries (OR 0.05, *p* < 0.001). *KIT*-altered melanoma showed the opposite pattern, occurring in 10 of 50 mucosal primaries (20.0%) and in only 1 of 19 cutaneous primaries (5.3%) (OR for mucosal vs. non-mucosal 2.65, *p* = 0.102). The *RAS*-altered subgroup tended to be more common in acral primaries (7/24, 29.2% vs. 10/84, 11.9% elsewhere; OR 3.05, *p* = 0.056), whereas the small *NF1*-altered subgroup (*n* = 7) and quadruple–wild-type tumors were distributed across all primary sites without significant enrichment.

TMB did not differ significantly across primary sites (*p* = 0.194). The median TMB was 3.9 mut/Mb in cutaneous (IQR, 2.3–7.1), 4.7 in acral (IQR, 2.6–5.5), 5.5 in mucosal (IQR, 3.3–7.5), and 3.1 in other primary sites (IQR, 1.9–5.9). TMB-high tumors were uncommon across all primary sites.

### 3.6. Multivariable Prognostic Analysis for PFS

To assess the independent prognostic effect of molecular subtype, univariable and multivariable Cox proportional hazards analyses were performed for PFS (Table 3). In the univariable analysis, a higher number of metastatic sites, *BRAF*-altered status (driven primarily by *BRAF* fusion), *KIT*-altered status, and mucosal primary site were significantly associated with shorter PFS, whereas sex, age, NLR, TMB-high status, and ICI regimen were not. The multivariable model included molecular subtype (quadruple-wild-type as reference, with *BRAF* V600 and *BRAF* fusion modeled as separate categories), age, number of metastatic sites, and TMB status. It was fitted in 108 patients with 75 PFS events, corresponding to eight estimated parameters and approximately 9.4 events per parameter. The primary site was not entered as an independent covariate because it was strongly associated with molecular subtype (*p* < 0.001) and was considered biologically related to subtype distribution rather than an independent prognostic factor.

After multivariable adjustment, *BRAF* fusion was independently associated with inferior PFS (HR 5.05, 95% CI 1.70–14.98, *p* = 0.003). *BRAF* V600 showed a numerically increased risk that did not reach statistical significance (HR 1.77, 95% CI 0.82–3.82, *p* = 0.149). *KIT*-altered status (HR 2.91, 95% CI 1.46–5.77, *p* = 0.002), and a higher number of metastatic sites (HR per additional site 1.42, 95% CI 1.10–1.83, *p* = 0.008) also remained independent adverse prognostic factors. *RAS*- and *NF1*-altered status were not independent predictors of PFS, and neither age nor TMB status retained statistical significance. In a sensitivity analysis additionally adjusting for prior adjuvant ICI, the associations for *BRAF* fusion and *KIT*-altered status were essentially unchanged. These associations were also robust in a Firth penalized model (Supplementary Materials Table S3). *KIT*-altered status remained an independent adverse prognostic factor in a model stratified by primary site (HR 3.21, 95% CI 1.57–6.55, *p* = 0.001). The proportional hazards assumption was satisfied, with no covariate showing a significant violation on testing of scaled Schoenfeld residuals (global *p* = 0.40). Because the number of OS events was limited, multivariable analysis was not performed for OS.

## 4. Discussion

In 135 patients with metastatic melanoma treated with first-line ICI, we observed an ORR of 37.8% and a median PFS of 6.2 months, with mucosal and acral subtypes together accounting for two-thirds of the cohort. Among 108 patients with paired NGS data, molecular subtype was significantly associated with PFS (log-rank *p* = 0.009), and *BRAF*- and *KIT*-altered status emerged as independent adverse prognostic factors after adjustment for clinical covariates. Sensitivity analysis revealed that *BRAF* fusion, rather than *BRAF* V600 SNVs, was the principal driver of the unfavorable outcome in the *BRAF*-altered subgroup. TMB-high status was uncommon and was not associated with improved outcomes. Together, these findings highlight features of metastatic melanoma in an East Asian population that differ substantively from those reported in Western melanoma cohorts and inform the prognostic value of molecular subtyping in this setting.

The efficacy of first-line ICI in our cohort is consistent with previously reported outcomes in Asian melanoma populations, in which the predominance of acral and mucosal subtypes is associated with lower response rates compared with Western cohorts. In KEYNOTE-006, treatment-naive patients with predominantly cutaneous melanoma achieved an ORR of approximately 42% and a median PFS of 8.4 months with pembrolizumab [7]. By contrast, Asian retrospective analyses of metastatic melanoma have generally reported ORRs in the range of 11–20% and a median PFS of 3–5 months, with acral and mucosal subtypes consistently showing inferior outcomes compared with cutaneous melanoma [5,22,23]. Our ORR of 37.8% and median PFS of 6.2 months fall between these two ranges, possibly reflecting more recent clinical practice, broader access to first-line ICI, and updated diagnostic and follow-up workflows.

The molecular landscape of our cohort reflects the distinct epidemiology and biology of melanoma in East Asia. Whereas Western melanoma cohorts typically harbor *BRAF* mutations in approximately half of patients and have a relatively low proportion of triple–wild-type tumors [13], our cohort exhibited a markedly different distribution: *BRAF*-altered tumors accounted for only 15.7% overall, *KIT*-altered tumors for 13.9%, and quadruple–wild-type tumors for nearly half (48.1%). This pattern was strongly shaped by primary site: *BRAF* alterations were heavily concentrated in cutaneous primaries and virtually absent in mucosal primaries, whereas *KIT* alterations and quadruple–wild-type tumors were enriched in mucosal and acral primaries.

One of the most clinically relevant findings was the unfavorable outcome of *BRAF*-altered patients on ICI, driven predominantly by *BRAF* fusions rather than canonical V600 mutations. This is notable because *BRAF* V600 status has generally not been associated with inferior anti-PD-1 outcomes in Western cutaneous melanoma [19]; in our cohort, the adverse signal instead tracked specifically with the *BRAF* fusion subset. More broadly, the overall ICI efficacy in our cohort may reflect its distinct mutational context. Western cutaneous melanomas typically arise in a UV-induced mutational landscape characterized by a median TMB of approximately 13 mut/Mb, which generates abundant neoantigens and renders them highly immunogenic [3,13]. In our cohort, by contrast, the median TMB was 4.7 mut/Mb, and even within cutaneous primaries the median TMB was only 3.9 mut/Mb. This low UV-driven mutational load may blunt the neoantigen-dependent immunogenicity that underlies ICI sensitivity, providing a biologic rationale for the lower overall ICI efficacy observed in this acral- and mucosal-predominant cohort.

The analysis separating *BRAF* V600 SNVs from *BRAF* fusions further refined this observation. Although both subgroups had inferior outcomes, the effect was disproportionately driven by *BRAF* fusions, which conferred a markedly elevated risk of progression (HR 5.05, 95% CI 1.70–14.98) and yielded no objective responses in any of the 4 patients in our cohort. *BRAF* fusions are a rare class of *BRAF* alterations that constitutively activate *MAPK* signaling through a fundamentally different mechanism than V600 SNVs, and the available evidence suggests they are refractory to *BRAF* inhibition and have been associated with inferior outcomes on ICI therapy [19,24]. Our data are consistent with these prior observations and suggest that *BRAF* fusion tumors are prognostically distinct from *BRAF* V600-mutant tumors and should not be assumed to share their ICI outcomes. This finding, however, is based on only four patients with a wide CI and should be regarded as hypothesis-generating pending validation in larger cohorts.

*KIT*-altered status was an independent adverse prognostic factor for PFS in our cohort (HR 2.91, 95% CI 1.46–5.77), with a median PFS of 4.8 months. *KIT* alterations are characteristic of acral and mucosal melanoma and are uncommon in Western cutaneous melanoma [14]. As a consequence, the prognostic impact of *KIT* alterations on ICI outcomes has been underexplored in pivotal trials. Tumors with *KIT* alterations typically lack a UV mutational signature and have a low TMB, which may contribute to reduced immunogenicity. Our findings suggest that *KIT*-altered melanoma represents a clinically meaningful subgroup with inferior outcomes on first-line ICI, supporting consideration of alternative strategies such as *KIT*-targeted therapies or combination regimens in this population [15].

TMB-high status was uncommon in our cohort (11 patients, 10.2% overall) and was not significantly associated with ICI outcomes, although this small subgroup limits the statistical power to detect such an association. This contrasts with Western melanoma cohorts in which TMB-high status, often defined by a UV-induced mutational signature, has been associated with superior response to PD-1 blockade [18]. The low TMB distribution observed in our cohort, including in cutaneous primaries, likely reflects a smaller contribution of UV-driven mutagenesis in our East Asian patient population, in which non-chronic sun damage pathways predominate [25]. These findings raise the possibility that TMB thresholds and predictive frameworks developed in Western melanoma populations may not translate directly to East Asian melanoma cohorts, and that additional or alternative biomarkers may be needed to guide ICI selection in this setting.

One important consideration applies to the prognostic associations reported here for *BRAF* fusion and *KIT* alterations. Because all patients in our cohort received first-line ICI and no non-ICI comparator was available, the associations we report are prognostic rather than predictive. They identify subgroups with poorer outcomes on ICI but cannot, by themselves, separate generally aggressive tumor biology from a reduction in benefit that is specific to immunotherapy. The inferior outcomes of *KIT*-altered and *BRAF* fusion tumors, for instance, may reflect a more aggressive natural history, consistent with their typically low, non-UV-related mutational burden and reduced immunogenicity, rather than selective resistance to ICI. Distinguishing these possibilities will require studies that include a non-ICI comparator or that formally test treatment-by-subtype interactions.

Several limitations should be acknowledged. As a single-center retrospective study, our findings are subject to the inherent biases of retrospective analyses, and external validation in independent East Asian cohorts is warranted. In addition, response assessments were performed by the treating physician according to RECIST 1.1 without independent or blinded central review, which may have introduced bias into the assessment of ORR and PFS. Follow-up duration was limited, particularly for OS outcomes, which precluded multivariable analysis for OS. Molecular profiling was performed using two tissue platforms (the TSO500 DNA/RNA assay and the Oncomine Comprehensive Assay Plus) and, in approximately one-fifth of the patients, plasma ctDNA. These platforms and media differ in their sensitivity for detecting gene fusions and copy-number alterations. All four *BRAF* fusions were identified on the TSO500 assay, which incorporates RNA-based fusion detection, whereas no *BRAF* fusion was detected among the 51 patients (47%) profiled by the Oncomine assay or ctDNA. Because fusion and copy-number detection can be less sensitive with these approaches, it is possible that some *BRAF* fusions were under-detected in this subset, and a small number of fusion-positive tumors may have been misclassified, for example, as quadruple-wild-type. Confirmation using uniform, fusion-sensitive tissue profiling in future studies would be valuable.

The subgroup sizes for *BRAF* fusion (*n* = 4) and *NF1*-altered (*n* = 7) tumors were small, limiting the precision of the corresponding effect estimates and calling for cautious interpretation. The cohort was predominantly treated with pembrolizumab, largely reflecting the convenience of its every-3-week dosing schedule. As both agents are anti-PD-1 monotherapies with comparable efficacy, this imbalance is unlikely to have materially affected the overall efficacy estimates; nevertheless, the direct comparison between the two agents is limited by the small number of nivolumab-treated patients (*n* = 19) and should be interpreted as exploratory.

Despite these limitations, our study has several notable strengths. We report a substantial cohort of metastatic melanoma in an East Asian population, with all patients receiving uniformly first-line ICI in the contemporary treatment era. Paired tumor or ctDNA NGS was available for the majority of patients (80.0%), enabling integrated efficacy and molecular analyses in this acral- and mucosal-predominant population. The analytic framework, including the hierarchical molecular subtype classification and the separation of *BRAF* V600 and *BRAF* fusion subgroups, allowed us to detect clinically meaningful effects that may be obscured when *BRAF* alterations are analyzed as a single class.

## 5. Conclusions

First-line ICI achieved meaningful efficacy in this East Asian metastatic melanoma cohort, with outcomes intermediate between earlier Asian cohorts and Western trials. Molecular subtype was strongly associated with PFS, and *BRAF*- and *KIT*-altered tumors, particularly those harboring *BRAF* fusions, were associated with inferior outcomes. The primary site-subtype distribution in our cohort was distinct. Together, these observations support the view that prognostic and predictive frameworks for metastatic melanoma derived from Western populations require recalibration when applied to East Asian patients. These findings call for prospective studies of subtype-specific treatment strategies and for the development of additional genetic and clinical biomarkers to refine prognostication and guide personalized treatment in East Asian metastatic melanoma.

## Figures and Tables

**Table 1:** Baseline characteristics (*n* = 135).

Characteristic	Value (*n* = 135)
Age, years, median (IQR)	64.7 (57.2–73.3)
Sex, *n* (%)	
Male	74 (54.8)
Female	61 (45.2)
ECOG performance status, *n* (%)	
0–1	133 (98.5)
2	2 (1.5)
Smoking status, *n* (%)	
Current smoker/Ex-smoker	78 (57.8)
Never smoker	57 (42.2)
Primary melanoma subtype, *n* (%)	
Cutaneous	25 (18.5)
Acral	31 (23.0)
Mucosal	58 (43.0)
Uveal	4 (3.0)
Other/Unknown	17 (12.6)
Disease stage, *n* (%)	
IV	135 (100.0)
M stage, *n* (%)	
M1a	67 (49.6)
M1b	24 (17.8)
M1c	37 (27.4)
M1d	7 (5.2)
Disease presentation, *n* (%)	
De novo	61 (45.2)
Recurrent	74 (54.8)
Metastatic site involvement, *n* (%)	
Lymph node	92 (68.1)
Lung	44 (32.6)
Soft tissue	32 (23.7)
Liver	30 (22.2)
Bone	26 (19.3)
Central nervous system	9 (6.7)
LDH, *n* (%)	
Normal	35 (25.9)
Elevated (>225 U/L)	33 (24.4)
Not available	67 (49.6)
NLR, *n* (%)	
NLR ≥3	35 (25.9)
NLR <3	95 (70.4)
Not available	5 (3.7)
ICI regimen, *n* (%)	
Pembrolizumab	116 (85.9)
Nivolumab	19 (14.1)
Molecular subtype (NGS subset, *n* = 108), *n* (%)	
* BRAF*-altered	17 (15.7)
* RAS*-altered	17 (15.7)
* NF1*-altered	7 (6.5)
* KIT*-altered	15 (13.9)
* Quadruple*–wild-type	52 (48.1)

Abbreviations: IQR, interquartile range; ECOG, Eastern Cooperative Oncology Group performance status; LDH, lactate dehydrogenase; NLR, neutrophil-to-lymphocyte ratio; ICI, immune checkpoint inhibitor; NGS, next-generation sequencing.

**Table 2:** Efficacy outcomes by first-line ICI regimen (*n* = 135).

Outcome	Pembrolizumab (*n* = 116)	Nivolumab (*n* = 19)	Overall (*n* = 135)
Best overall response, *n* (%)			
Complete response	3 (2.6)	3 (15.8)	6 (4.4)
Partial response	43 (37.1)	2 (10.5)	45 (33.3)
Stable disease	32 (27.6)	9 (47.4)	41 (30.4)
Progressive disease	38 (32.8)	5 (26.3)	43 (31.9)
Response rates			
Objective response rate, *n* (%)	46 (39.7)	5 (26.3)	51 (37.8)
Disease control rate, *n* (%)	78 (67.2)	14 (73.7)	92 (68.1)
Survival, months (95% CI)			
Median PFS	6.2 (3.7–9.4)	9.9 (2.2–NR)	6.2 (4.0–9.6)
Median OS	NR (28.4–NR)	NR (14.1–NR)	NR (46.6–NR)

Abbreviations: ICI, immune checkpoint inhibitor; PFS, progression-free survival; OS, overall survival; CI, confidence interval; NR, not reached.

**Table 3:** Univariable and multivariable Cox proportional hazards analyses for progression-free survival (*n* = 108).

Covariate	*n*	Univariable Analysis			Multivariable Analysis		
		HR	95% CI	*p*	HR	95% CI	*p*
Sex							
Female	47	1.00 [Ref]					
Male	61	0.97	0.62–1.54	0.912			
Age (per year)	108	0.99	0.97–1.01	0.241	0.99	0.97–1.01	0.470
Primary site							
Cutaneous	19	1.00 [Ref]					
Acral	24	0.54	0.28–1.07	0.076			
Mucosal	50	0.38	0.21–0.68	<0.001			
Other	15	0.45	0.20–1.01	0.054			
Number of metastatic sites (per +1)	108	1.42	1.10–1.82	0.007	1.42	1.10–1.83	0.008
NLR							
<3	78	1.00 [Ref]					
≥3	26	1.07	0.62–1.85	0.810			
TMB status							
TMB-low	97	1.00 [Ref]			1.00 [Ref]		
TMB-high	11	0.86	0.39–1.87	0.703	0.99	0.42–2.31	0.983
ICI regimen							
Pembrolizumab	95	1.00 [Ref]					
Nivolumab	13	0.81	0.39–1.68	0.565			
Molecular subtype							
Quadruple–wild-type	52	1.00 [Ref]			1.00 [Ref]		
*BRAF V600*	13	2.05	0.97–4.36	0.061	1.77	0.82–3.82	0.149
*BRAF* fusion	4	5.20	1.77–15.26	0.003	5.05	1.70–14.98	0.003
*RAS*-altered	17	1.49	0.76–2.94	0.245	1.37	0.69–2.73	0.368
*NF1*-altered	7	1.26	0.49–3.23	0.638	1.28	0.49–3.34	0.617
*KIT*-altered	15	2.90	1.47–5.71	0.002	2.91	1.46–5.77	0.002

Abbreviations: HR, hazard ratio; CI, confidence interval; Ref, reference category; NLR, neutrophil-to-lymphocyte ratio; TMB, tumor mutational burden; ICI, immune checkpoint inhibitor.

## Data Availability

The data presented in this study are available on request from the corresponding author. The data are not publicly available due to privacy and ethical restrictions.

## Supplementary Materials

The following supporting information can be downloaded at: https://www.mdpi.com/article/10.3390/cancers18172768/s1, Figure S1: Study population; Figure S2: Molecular subtype distribution across primary sites; Table S1: Frequency of co-occurring genomic alterations by molecular subtype; Table S2: Distribution of molecular subtypes across primary tumor sites; Table S3: Multivariable Cox proportional hazards analyses for progression-free survival: primary model, sensitivity analysis adjusting for prior adjuvant ICI, and Firth penalized model.
