# Supplementary material for: The Impact of Molecular Characteristics on the Efficacy of Frontline Immune Checkpoint Inhibitor Therapy in Patients with Metastatic Melanoma
Source: Cancers (Basel). 2026 Aug 26;18(17):2768. doi: 10.3390/cancers18172768 (PMC13564568; doi:10.3390/cancers18172768)
Supplement: Supplementary file 1 [file cancers-18-02768-s001.zip › cancers-4441407-supplementary.pdf]

Figure S1. Study population

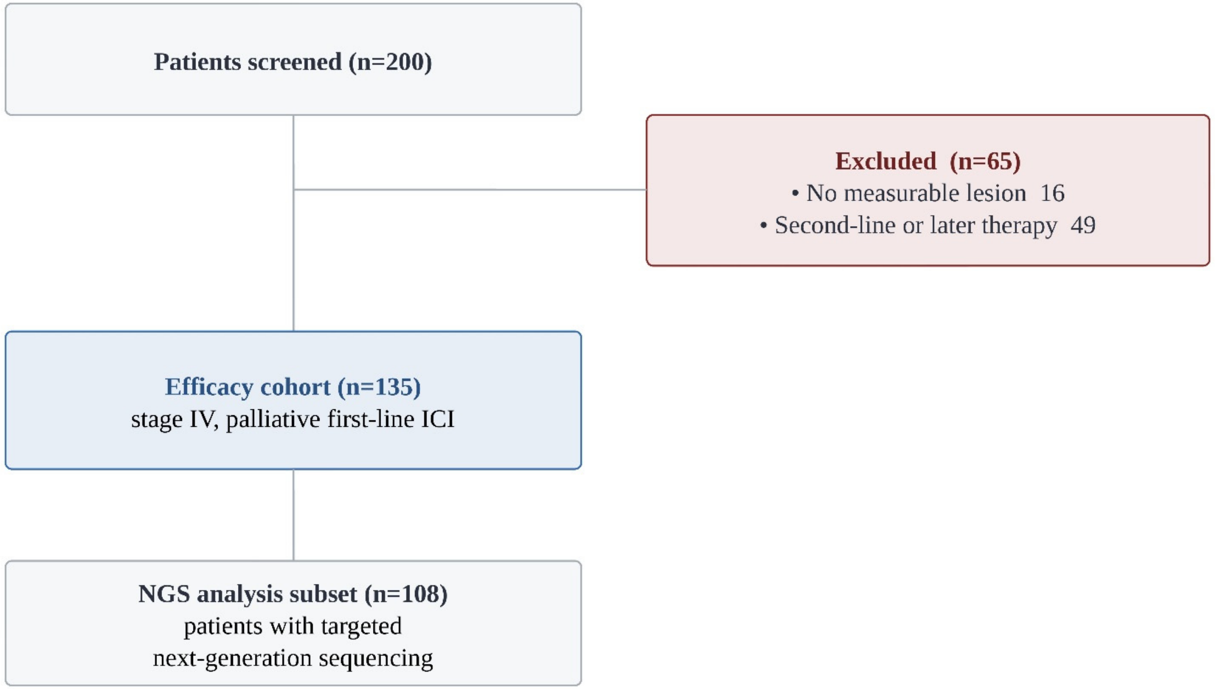

Figure S2. Molecular subtype distribution across primary sites

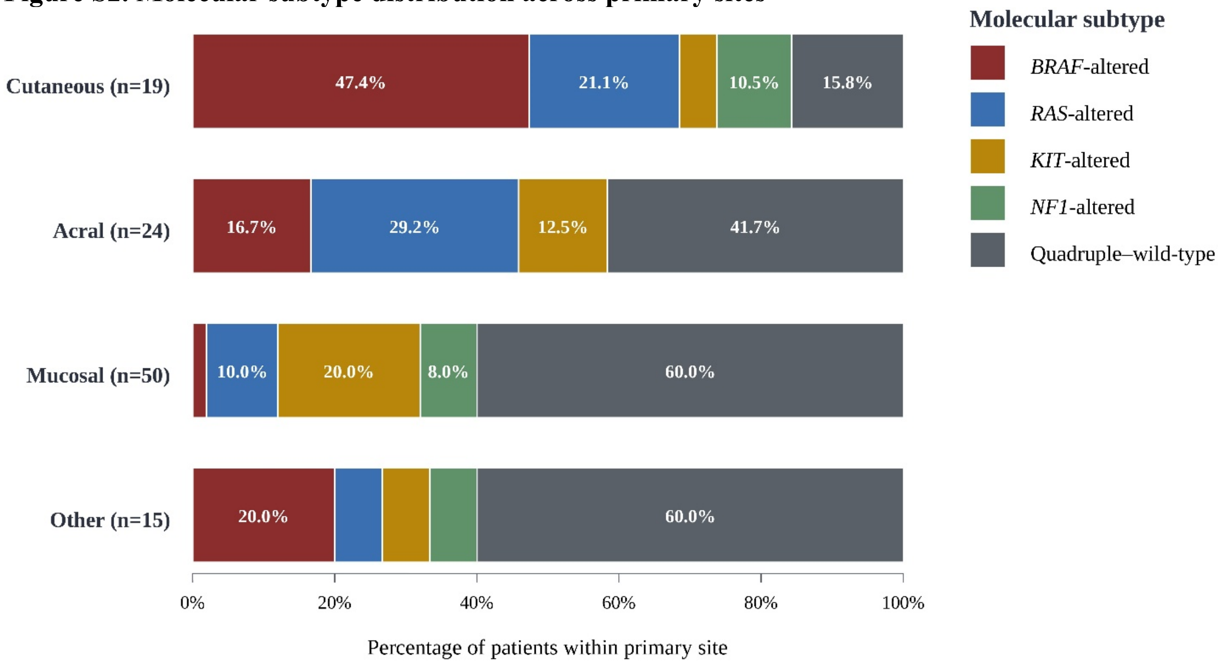

Supplementary Table S1. Frequency of co-occurring genomic alterations by molecular subtype (n=108)

**Molecular subtype**	***CDKN2A***	***TERT***	***PIK3CA***	***PTEN***	***TP53***
BRAF V600 (n=13)	3 (23.1%)	4 (30.8%)	0	1 (7.7%)	0
BRAF fusion (n=4)	0	0	0	0	0
RAS-altered (n=17)	4 (23.5%)	2 (11.8%)	1 (5.9%)	2 (11.8%)	0
NF1-altered (n=7)	4 (57.1%)	1 (14.3%)	1 (14.3%)	0	1 (14.3%)
KIT-altered (n=15)	3 (20.0%)	1 (6.7%)	0	0	0
Quadruple-wild-type (n-52)	3 (5.8%)	1 (1.9%)	4 (7.7%)	2 (3.8%)	1 (1.9%)
**Overall (n=108)**	**17 (15.7%)**	**9 (8.3%)**	**6 (5.6%)**	**5 (4.6%)**	**2 (1.9%)**

Supplementary Table S2. Distribution of molecular subtypes across primary tumor sites (n=108)

Molecular subtype	Cutaneous (n=19)	Acral (n=24)	Mucosal (n=50)	Other (n=15)	Total (n=108)
BRAF V600	8 (42.1%)	2 (8.3%)	0	3 (20.0%)	13 (12.0%)
BRAF fusion	1 (5.3)	2 (8.3%)	1 (2.0%)	0	4 (3.7%)
RAS-altered	4 (21.1%)	7 (29.2%)	5 (10.0%)	1 (6.7%)	17 (15.7%)
NF1-altered	2 (10.5%)	0	4 (8.0%)	1 (6.7%)	7 (6.5%)
KIT-altered	1 (5.3%)	3 (12.5%)	10 (20.0%)	1 (6.7%)	15 (13.9%)
Quadruple-wild-type	3 (15.8%)	10 (41.7%)	30 (60.0%)	9 (60.0%)	52 (48.1%)

Supplementary Table S3. Multivariable Cox proportional hazards analyses for progression-free survival: primary model, sensitivity analysis adjusting for prior adjuvant ICI, and Firth penalized model (n=108)

**Covariate**	**n**	**Primary multivariable model**			**Sensitivity model (+ Prior adjuvant ICI)**			**Firth penalized model**		
		**HR**	**95% CI**	***p***	**HR**	**95% CI**	***p***	**HR**	**95% CI**	***p***
**Age (per year)**	108	0.99	0.97–1.01	0.470	0.99	0.97-1.01	0.443	0.99	0.97-1.01	0.463
**Number of metastatic sites (per +1)**	108	1.42	1.10–1.83	0.008	1.41	1.09-1.82	0.009	1.42	1.10-1.83	0.007
**TMB status**										
TMB-low	97	*1.00 [Ref]*			*1.00 [Ref]*			*1.00 [Ref]*		
TMB-high	11	0.99	0.42–2.31	0.983	0.98	0.42-2.29	0.966	1.04	0.46-2.38	0.918
**Molecular subtype**										
Quadruple–wild-type	52	*1.00 [Ref]*			*1.00 [Ref]*			*1.00 [Ref]*		
BRAF V600	13	1.77	0.82–3.82	0.149	1.91	0.87-4.18	0.105	1.82	0.85-3.86	0.122
BRAF fusion	4	5.05	1.70–14.98	0.003	5.08	1.71-15.07	0.003	5.43	1.93-15.29	0.001
RAS-altered	17	1.37	0.69–2.73	0.368	1.39	0.70-2.76	0.352	1.40	0.71-2.75	0.330
NF1-altered	7	1.28	0.49–3.34	0.617	1.28	0.49-3.33	0.621	1.38	0.55-3.47	0.493
KIT-altered	15	2.91	1.46–5.77	0.002	3.13	1.56-6.26	0.001	2.93	1.49-5.76	0.002
**Prior adjuvant ICI**										
No	104	-			1.00 *[Ref]*			-		
Yes	4	-			0.50	0.12-2.12	0.347	-		

The primary model is identical to the multivariable analysis in Table 3. The sensitivity model adds prior adjuvant ICI as a covariate. The Firth penalized model addresses potential small-sample and separation bias; 95% CIs are penalized Wald intervals. Prior adjuvant ICI was not included in the primary or Firth models.

Abbreviations: HR, hazard ratio; CI, confidence interval; Ref, reference category; TMB, tumor mutational burden; ICI, immune checkpoint inhibitor.
